# Feasibility, acceptability and validity of electronic adherence monitoring among adolescents in Zimbabwe: a mixed methods study

**DOI:** 10.1186/s44263-026-00248-z

**Published:** 2026-02-10

**Authors:** Nyasha V. Dzavakwa, Constance RS. Mackworth-Young, Palwasha Y. Khan, Hilda A. Mujuru, Mazvita Paradza, Marshall T. Chiwodza, Panashe Bluck, Nicol Redzo, Tsitsi Bandason, Katharina Kranzer, Rashida A. Ferrand, Victoria Simms

**Affiliations:** 1 Biomedical Research and Training Institute, 10 Seagrave Road, Avondale, Harare, Zimbabwe; 2 Department of Infectious Disease Epidemiology and International Health, London School of Hygiene and Tropical Medicine, London, UK; 3 Department of Global Health and Development, London School of Hygiene and Tropical Medicine, London, UK; 4 Clinical Research Department, London School of Hygiene and Tropical Medicine, London, UK; 5 Department of Child, Adolescent and Women’s Health, University of Zimbabwe, Harare, Zimbabwe; 6 Institute of Infectious Diseases and Tropical Medicine, Ludwig-Maximilians-Universität München, University Hospital, Ludwig-Maximilians-Universität München, Munich, Germany

**Keywords:** Electronic monitoring devices, Adolescents, Validity, Feasibility, Acceptability, Adherence-monitoring

## Abstract

**Background:**

Electronic monitoring devices (EMDs) may provide an objective method for assessing medication adherence. However, evidence on their validity compared to other adherence measures, their functional feasibility, and their acceptability, especially among adolescents, remains limited. Adolescents face multifaceted adherence challenges, yet there is a lack of evidence to inform the use of digital tools for adherence monitoring and support in this age group, particularly in low-income settings. We assessed feasibility, acceptability, and validity of an EMD among adolescents enrolled in the multi-country clinical trial VITALITY.

**Methods:**

An explanatory sequential mixed methods study was embedded within the Zimbabwean site of the VITALITY trial, a randomised controlled trial evaluating weekly vitamin D supplementation on bone health in adolescents living with HIV. A random sample of 97 participants (aged 11–19 years) was provided an EMD for 24 weeks to monitor adherence to vitamin D or placebo. Validity was assessed by correlating EMD-measured adherence with serum vitamin D levels. Feasibility of the EMD was investigated through records of battery duration, network connectivity, and EMD malfunction. Seventeen participants were purposively selected for qualitative exit interviews to explore acceptability and reasons for discordance between adherence measured through the EMD and pill count.

**Results:**

Ninety-seven participants, median age 16.7 (interquartile range13.5, 18.7) years, 50 (51.5%) female were provided with the EMD for a median of 24 (range 22–25) weeks. There was a strong positive correlation between EMD-measured adherence and week-48 vitamin D levels (β = 0.63, 95% CI: 0.42–0.85; R^2^ = 0.42; *p* < 0.001), supporting the validity of EMD data as a proxy for medication intake. The EMD functioned reliably despite intermittent network coverage, and no major malfunctions were reported. Adolescents found the EMDs highly acceptable due to their ease of use, discretion, and perceived motivational benefits.

**Conclusions:**

This study demonstrates that EMDs are valid, feasible, and acceptable tools for monitoring adherence among adolescents in low-income settings. These findings support the potential for broader use of EMDs to promote and monitor adherence to long-term treatments in adolescents beyond research settings.

**Trial registration:**

Pan African Clinical Trials Registry (PACTR), PACTR202009897660297.

**Supplementary Information:**

The online version contains supplementary material available at 10.1186/s44263-026-00248-z.

## Background

Adherence to prescribed medications is a recognised challenge in adolescents with chronic conditions [1, 2]. Sub-optimal adherence to treatment results in an increased risk of disease complications, increased use of hospital services, unnecessary changes in regimen, and an overall reduction in quality of life [2, 3]. Thus, accurate assessment of adherence is important to identify those at risk of non-adherence to provide adherence support [1, 4].

Assessment of adherence remains a persistent challenge in both research and clinical practice due to lack of valid implementable measurement methods [5]. Indirect measures such as pill count and self-report are subject to social desirability bias and can overestimate adherence [5]. Pharmacy refill is another commonly used indirect measure of adherence that assesses the timeliness of medication pick-up [6]. Although pharmacy refill does not confirm ingestion and therefore provides only a crude estimate of adherence, it has been shown to be strongly associated with viral suppression among individuals receiving antiretroviral therapy (ART) [7, 8]. Direct measures of adherence such as measurement of a drug or its metabolite in body fluids provide evidence of drug ingestion, but they cannot show patterns of adherence [9, 10]. The amount of drug detected in the body may vary across different individuals regardless of adherence behaviour due to differences in metabolic and physiological states [5, 11].

Electronic monitoring devices (EMDs) may be an alternative method of assessing adherence to measuring drug levels, pill count, self-report or refill [12]. EMDs contain a microprocessor that utilizes mobile network technology to record the date and time when a pillbox is opened, an inhalation device is actuated, or eye drops are dispensed [13]. This information can be accessed by healthcare providers and provides real-time data on medication adherence and timely detection of lapses in adherence [5, 14]. However, like all adherence measures, EMDs are susceptible to certain sources of bias. Periods of non-use or incorrect use can result in misclassification of adherence [15]. For example, some individuals may remove multiple pills at once to take later (pocket dosing), resulting in underestimated adherence despite appropriate medication usage [15, 16]. Conversely, opening the device without ingesting medication (“curiosity openings”) may lead to overestimated adherence [15, 17].

EMDs have been used to measure adherence in children and in adults, but evidence among adolescents, particularly in low-income settings, is lacking [18, 19]. To address this gap, we evaluated the feasibility, acceptability, and validity of an EMD for measuring adherence to weekly vitamin D supplementation among adolescents in Zimbabwe.

## Methods

### Study design and participant selection

This explanatory sequential mixed methods study was prospectively designed and embedded within the Zimbabwean site of a multi-country, individually randomised, double-blind, placebo-controlled trial VITALITY (PACTR202009897660297, https://pactr.samrc.ac.za/TrialDisplay.aspx?TrialID=12318) investigating the impact of high-dose vitamin D and calcium carbonate supplementation on bone health among adolescents living with HIV [20]. The embedded mixed methods study was designed to assess the feasibility, acceptability, and validity of an EMD among adolescents participating in the VITALITY trial.

The VITALITY trial protocol has been published [21]. The primary outcome of the VITALITY trial was total body less-head bone mineral density Z score, and the secondary outcome was lumbar spine bone mineral apparent density Z score, both measured by dual-energy X-ray absorptiometry; these outcomes have been reported elsewhere [22]. Briefly, participants were adolescents aged 11–19 years with perinatally acquired HIV attending outpatient clinics in Lusaka, Zambia and Harare, Zimbabwe. Eligible participants at both sites had been on ART for at least 6 months before enrolment into the VITALITY trial. The Lusaka site was the Women and Newborn Hospital at the University Teaching Hospital, a tertiary hospital within the capital city of Lusaka, which has a population of approximately 3.3 million people [23]. The Harare site was the Children’s Hospital at Sally Mugabe Central Hospital, a tertiary hospital located within a city of about 2.1 million people [24]. These two are the main public sector hospitals in the two cities, providing specialist paediatric and adolescent HIV services, and serve as teaching and referral hospitals. Participants were randomised 1:1 to receive weekly high-dose vitamin D (20,000 international units) plus daily calcium carbonate, or identical in shape and test placebo for 48 weeks (henceforth referred to as trial drugs). Participants had 12-weekly study follow-up visits to dispense trial drugs and to monitor adherence and side-effects [21]. Trial participants were informed that the once-weekly trial drugs should be taken on the same day of the week.

For this nested study, inclusion was restricted to adolescents enrolled in the Zimbabwe cohort of the parent VITALITY trial, leveraging the existing cohort of adolescents living with HIV. Participants were eligible if they had enrolled between 30 August and 23 November 2021 and remained under active follow-up after 24 weeks. Exclusion criteria included enrolment into the VITALITY trial at the Zambia site, enrolment outside the specified period, and withdrawal or exit from the parent trial before the week 24 visit.

Quantitative data collection was undertaken first to assess the feasibility of EMD-measured adherence; this was followed by phenomenological qualitative interviews with a subset of participants to explore acceptability [20, 25]. This approach allowed the quantitative findings to be contextualized through participants’ lived experiences.

Between 14 February and 21 May 2022, 97 participants were selected by simple random sampling from the 422 VITALITY trial participants enrolled in Zimbabwe to use an EMD for 24 weeks. Simple random sampling was conducted using a random number generator in Stata, with the randomization list generated by a statistician. The sample size was determined by the availability of 100 EMDs, which had been purchased specifically for the study. Due to cost constraints, no additional EMDs were procured. The final 97 participants included 50 receiving vitamin D and 47 receiving placebo. We investigated the validity of vitamin D adherence measured through the EMD against a biological marker of vitamin D status (25 hydroxy vitamin D – 25(OH)D) measured 48 weeks after initiation of trial drugs. We also assessed which adherence measure, i.e., pill count or the EMD, showed stronger correlation with 25(OH)D levels, focusing this analysis only on participants taking vitamin D. We also assessed whether there was concordance between the total number of tablets taken as measured by pill count and the total number of EMD opening events in all 97 participants receiving either vitamin D or placebo. Feasibility of the EMD was assessed by recording battery duration, EMD malfunction or loss of network connectivity issues, and how these issues were addressed.

Seventeen participants were purposively selected for qualitative exit interviews to ensure diversity in age (early adolescence 11–13 years; middle 14–16 years; late 17–19 years), sex, and adherence patterns as recorded by the EMD (high, moderate and low). Qualitative interviews explored adolescents’ acceptability and experiences of using the EMD and reasons for discordance between adherence measured through the EMD and pill count. The qualitative component was informed by a constructivist paradigm, which assumes that meaning is co-constructed through participants’ lived experiences and researcher–participant interaction. This paradigm informed the use of semi-structured interviews incorporating both open- and closed-ended questions alongside a deductive coding approach based on pre-defined research questions.

### Electronic monitoring device

The Wisepill RT2000 electronic medication dispenser (Wisepill Technologies, Cape Town, South Africa) can fit up to twenty medium-sized tablets (Additional file 1: Fig. S1), [26]. Every time it is opened, it records and saves the date and time of opening. This information is transmitted to a secure cloud-based server using roaming mobile cellular technology [26]. A patient’s medication intake schedule is registered on the server, and adherence data can be reviewed through accessing real-time online adherence calendars (Additional file 2: Fig. S2) that can be downloaded as a report. The Wisepill has a battery life of up to 6 months. A signal report (heartbeat) is sent to the cloud-based server daily, reporting battery life and transmission signal strength, regardless of EMD opening events.

### Study procedures

Study participants were given the EMD filled with vitamin D/placebo at their week 24 trial follow-up visit and were shown a video on how to use the device at home. Participants were informed that the EMD would record how they were taking their drugs at home but that they would not receive any feedback on the adherence recorded through the EMD during the study period. The adherence calendar was only shown to participants at the end of the study period during the qualitative exit interviews.

At enrolment into this study, each participant had their once-weekly trial drugs intake schedule registered on the cloud-based server. The intake schedule was created based on the day of the week the participant was expected to take the drugs (Additional file 2: Fig. S2). The study team was blinded to participants’ adherence recorded through the EMD and did not provide feedback on adherence to study participants (which could have interfered with trial adherence support procedures).

Trial drugs were filled into the EMD at each subsequent 12-weekly VITALITY visit with a maximum follow-up of 48 weeks, and participants’ adherence to the trial drugs was assessed through pill count. All EMDs were fully charged during the visit, and network connectivity was checked. Email notifications on low battery status (battery level 21–30%) triggered recharging of the EMD. If network connectivity was lost during follow-up, participants were asked to bring the EMD to the clinic to upload data to the cloud server and issue a new EMD.

### Data collection

Sociodemographic data and pill counts were collected as part of the parent VITALITY trial. Announced pill counts were conducted during the scheduled 12-weekly follow-up visits at the VITALITY trial clinics and were performed by the VITALITY research field staff. Data on feasibility was recorded in a field project diary and included network connectivity issues and how these were addressed, low battery status, malfunctioning of devices and any other challenges. In the parent VITALITY trial, 25(OH)D levels were measured at enrolment (week 0), week 48, and week 96. For this sub-study, EMDs were deployed during the final 24 weeks of the 48-week trial drug supplementation period. Adherence over this period was validated against 25(OH)D levels measured at week 48. At the week 48 visit, 25(OH)D levels were assessed in all participants, and data on adherence and heartbeats recorded by the EMDs were downloaded from the server.

In-depth interviews were conducted at the trial clinic at the week-48 study visit. Participants were approached face-to-face. Interviews were conducted by two trained interviewers, MP (female) and PB (male). MP was also involved in recruiting participants for this study and had prior engagement with them, which may have facilitated rapport but could also introduce potential bias due to familiarity with participants. PB was an independent researcher not involved in this study’s activities, reducing the risk of bias from prior participant interaction. Participants were aware that interviews were conducted by MP and PB to explore the acceptability of EMDs and reasons for discordance between adherence measured through the EMD and pill count.

A semi-structured interview guide was used, containing both open- and close-ended questions to encourage participants to describe their experiences in their own words (Additional file 3).

The interviews explored acceptability and participants’ experiences of using the EMD, including ease of use and storage, privacy related to storing tablets, perceptions of being monitored, challenges encountered, and reasons for discordance between adherence measured through the EMD and pill count. The guide was pilot tested with two adolescent youth workers not included in the study to ensure clarity and appropriateness of the questions. Example questions included: *“How did you find using the EMD during the study?”*, *“Did you experience any challenges when using the device? What were these challenges?”*, and *“What might explain differences between what the pill count showed and what the device recorded?”.*

Interviews were conducted in English or Shona, based on the participant’s language preference and lasted between 12 and 30 min. Although a guardian was present, interviewers emphasized confidentiality, reassured participants that there were no right or wrong answers and encouraged them to speak freely about their experiences. No participants refused to participate. All interviews were audio-recorded using portable digital audio recorders, transcribed verbatim, and translated into English. Those conducted in Shona by the research team did not use any. Software-assisted translation tools, and field notes were taken during the interviews.

### Data analysis

Quantitative analysis was performed using STATA version 18.0. Descriptive statistics—mean, median, and proportions—were used to summarize participants’ characteristics and data on feasibility.

Adherence to trial drug measured through the EMD and pill count was analyzed as continuous variables. Adherence to trial drugs measured by EMD was defined as the proportion of total doses taken relative to the total expected doses for the time the participant was in the study. Doses taken were defined as EMD opening events that occurred on the scheduled intake day ± 24 h. For adherence measured by pill count, the number of pills given to the participant, minus the number returned, was calculated as a percentage out of expected doses. 25(OH)D levels were presented as continuous and categorical variables using a pre-specified cut-point of ≥ 75 nmol/to define sufficiency [27].

Univariable linear regression was used to assess the relationship between 25(OH)D levels and adherence measured through the EMD for participants taking vitamin D only, and the relationship between 25(OH)D levels and pill count for participants receiving vitamin D only. In addition, we used the Spearman’s rank correlation coefficient, a non-parametric measure of the strength and direction of a monotonic association between two variables, to examine concordance between the total number of tablets taken as measured by pill count and the total number of EMD opening events over the study period (both trial arms included) [28]. This approach was selected because both adherence measures were not normally distributed. Correlation coefficients were interpreted as follows: values close to + 1 indicated strong positive concordance, values near 0 indicated little or no association, and values close to –1 indicated strong negative concordance [29]. Results were reported with 95% confidence intervals [29].

To assess the feasibility of EMD use, data were analyzed on the regularity of heartbeat signals sent from the EMD to the server, the duration of connectivity gaps, and any data loss. Feasibility was quantified using descriptive statistics, including the proportion of person-days with a recorded heartbeat, and the median number of consecutive days without a heartbeat signal. Challenges experienced during device use were also summarized descriptively.

Qualitative data was analyzed using thematic analysis. Following familiarization with the data, all transcripts were imported into NVivo-14 for coding. A deductive coding approach was used based on pre-defined research questions around acceptability and reasons for discordance between adherence measured by pill count and the EMD. Initial coding was performed by NVD (academic clinician) and reviewed by CMY. Codes were exported into an Excel spreadsheet and grouped to develop themes. Codes and themes were iteratively reviewed, revised, redefined, renamed, and regrouped through discussions between NVD, CMY, and VS (Additional file 4). An analytic memo was written by NVD, with input from CMY and VS, to bring together the data under the key themes and to draw out the main findings (Additional file 4). Qualitative data were reported according to the consolidated criteria for reporting qualitative research (Additional file 5) [30].

## Results

### Participant characteristics

The 97 enrolled participants (50 (51.5%) female, median age 16.7 (interquartile range (IQR): 13.5, 18.7) years) used the EMD for a median 24 (range 22–25) weeks. Half of the participants (*n* = 50, 51.5%) were from the intervention arm (taking vitamin D) of VITALITY (Table 1). In the 24 weeks preceding enrolment into this study, median adherence to trial drug, as measured through pill count, was 104.3 (IQR 100, 109.1) %.

**Table 1:** Sociodemographic and clinical characteristics of adolescents (*n* = 97) enrolled in this sub-study

Characteristic		*n*	%
Sex	Male Female	47 50	48.5 51.5
Age, years	Median (IQR)	16.7 (13.5, 18.7)	
VITALITY treatment arm	Receiving vitamin D Receiving placebo	50 47	51.5 48.5
Enrolled in school	Yes No	86 11	88.7 11.3
Current education level (*n* = 86)	In primary school In secondary school No information	33 48 5	40.7 59.3 5.8
Orphanhood	Both parents alive One or both parents deceased Don’t know	38 55 4	39.2 56.7 4.1
Primary caregiver	Mother Father Other	60 7 30	61.9 7.2 30.9
HIV viral load at enrolment into VITALITY	< 60 copies/ml 60–999 copies/ml ≥ 1000 copies/ml	84 3 10	86.6 3.1 10.3

*IQR* interquartile range

### Concordance of 25 hydroxy vitamin D with EMD and pill count adherence

To examine how well the EMD reflected actual intake, we first assessed the relationship between EMD-measured adherence and 25(OH)D levels and compared this with pill count adherence.

**Fig. 1:**
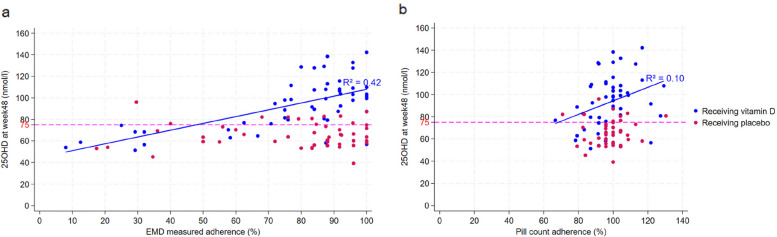
Graphs showing the relationship between 25 hydroxy vitamin D and EMD and pill count adherence in participants taking vitamin D

Among participants taking vitamin D, adherence at week 48 measured by pill count was higher than that measured by EMD with a median of 100% (IQR 91.7, 104.2%) vs median 87% (IQR 70.8, 100%), respectively. In linear regression, EMD-measured adherence was associated with vitamin D levels at week 48 (β = 0.63, 95% CI 0.42—0.85, R^2^ = 0.42, *p* < 0.001*)* but pill count adherence was not (β = 0.61, 95% CI 0.09—1.13, R^2^ = 0.10, *p* = 0.22; Fig. 1). The distribution of pill count adherence was also narrower compared to that of EMD-measured adherence.

### Concordance between EMD opening events and pill count

Having assessed agreement with the biological marker, we next compared the total number of EMD openings with tablets taken by pill count to further evaluate concordance between the two adherence measures.

**Fig. 2:**
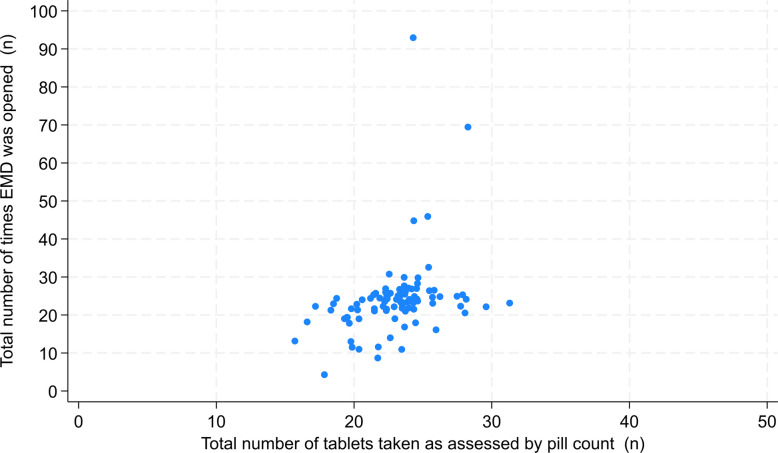
Concordance of total number of tablets taken assessed by pill count and total EMD opening events in all participants who used an EMD

There was a positive correlation (Spearman’s ρ = 0.53, 95% CI 0.37–0.69, *p* < 0.001) between EMD opening events and tablets taken by pill count (Fig. 2). However, some discordances were observed: six participants opened the EMD more times (maximum *n* = 93) than the number of tablets they took as assessed by pill count, while 18 participants opened the EMD fewer times (minimum *n* = 6) than the number of tablets they were reported to have taken by pill count. Qualitative data provided insights into the reasons for these discrepancies.

### Feasibility of EMDs

In addition to assessing validity, we evaluated the feasibility of EMD use, including device functionality, connectivity, and data transmission.

Overall, the EMD was feasible to use due to limited challenges reported, high regularity of heartbeat transmissions, and no loss of data, even when connectivity was disrupted. On 90% of person-days, the EMD sent a heartbeat to the server; the median duration without sending a heartbeat was 2 (IQR 1.3) days, including among participants who had visited areas outside of Harare with low mobile cellular network. Loss of network connectivity was the reason for not sending heartbeats. The longest gap between heartbeats was 86 days and was recorded on one EMD. While no information regarding corrective or preventive action was recorded about this device, no data was lost. Challenges experienced while using the EMD are summarized in Table 2.

**Table 2:** Issues arising with the use of electronic monitoring devices

Issue	Comment
EMDs that lost network connectivity	Seven EMDs lost communication with the server for more than 14 days, of which six had data manually uploaded, and the EMDs were swapped out with a working one. For one EMD, no information was recorded on the manual upload of data
Data loss	No data lost as the EMD can record and save all events even when offline
EMDs with low battery status	Seven EMDs reported a low battery (battery level 21–30%) after a median 19 weeks of use and these were brought back for recharging One EMD reported a critically low battery (battery level ≤ 20%) after 24 weeks of use. This device was not brought back for recharging as this event was reported three days before participant completed the study
EMDs lost/destroyed	No EMDs were lost One EMD was destroyed in a house fire and was damaged beyond repair
Challenges with EMD use or dispenser malfunction	None reported

*EMDs* electronic monitoring devices

### Acceptability of EMDs

To complement the quantitative findings, qualitative interviews were used to explore participants’ experiences with using the EMD.

Participants generally expressed satisfaction with using the EMD and valued its role in supporting medication-taking. However, they also highlighted a small number of challenges with usage.

#### Ease of use

Most participants expressed that the EMD was “*easy to use*”. They reported that it “*was never a problem to open and close the device*” (Male, 12 years). The instruction video that they watched at study enrolment was felt to be clear and helpful for using the EMD at home.

*“There was a time you made us watch a video on how to use the pill box. So, I understood there (how to use the EMD) when I was watching the video.”* (Female, 13 years).

#### Safe for storage of medication

Participants felt that EMDs were useful in keeping their tablets safe from environmental exposures and physical damage and reported that *“… the device was tightly closed such that my pills were not affected by air or wind”* (Female, 19 years).

#### Maintained privacy and was discreet

Older adolescents (16 years and above) felt that the EMD was useful for keeping their medication private, as it could easily pass for a power bank.

*“I liked the device, it helped me hide my medications… you can move around with it, while holding it in your hands a person may think it’s a power bank or even a phone…”* (Female, 18 years).

While the consensus was that the EMD was discreet, a couple of participants said they were not comfortable using it in public, as they feared it would attract attention from people.

*“When you open it, a light turns on. So, people would really question that, how come it lights? So, in public, you can’t take it out, because people may not understand… and end up seeing your pills”* (Male, 19 years).

One participant expressed that they found it difficult to move around with the EMD because of the size.

*“The device is not easy to carry around; it is big. With other containers you can take a tissue, remove the pills, wrap them up, and carry them around, but the device is very big, and you are not supposed to remove your pills… it’s difficult in that regard.”* (Female, 16 years).

#### Perceptions of being monitored

Participants discussed finding the EMD helpful in improving adherence, in large part because the perception of being monitored helped motivate a change in their adherence behaviour.

*“The good thing about this device is that it records me and reminds me to take my medication. I would say to myself, if I skip today, they will see me, so I would go and take my tablet.”* (Male, 12 years).

### Reasons for discordance between EMD opening events and pill count

Opening of EMD by other family members: Some participants reported that family members opened the EMD because they were curious.

“…where I opened the box outside the days (points to the adherence calendar), I can say that my siblings after seeing the box they started liking opening it. They were fascinated with the box and the light that came on when the device was opened and maybe also the pills themselves.” (Male, 19 years).

Use of EMD for other medications: A few participants highlighted that they opened the EMD every day because they also kept their antiretroviral therapy tablets in the EMD.

“At times when I was travelling, I would also put my ARVs (which I take every day) in the device as well and this helped me a lot” (Male, 20 years).

Took extra tablets at home: A few participants mentioned that they had extra tablets at home prior to being enrolled in this study. They took those tablets first before they started using the ones in the EMD.

“Before we started there were other tablets, I had at home which were not put in the device. I was told to finish those ones before the ones in the device.” (Female, 14 years).

Misunderstanding about the purpose of the EMD: A few participants indicated that they did not use the EMD because they did not understand its purpose and thought the EMD was meant for storage of tablets.

“I opened the device three or four times because I did not understand what the purpose of the device was, I thought that it was something used to store pills. I took out the tray (AM-PM plastic container) inside and took my tablets from there, I thought it was the same using the device and taking pills from the tray” (Male, 19 years).

## Discussion

This study found that adherence measured through an EMD was a more reliable predictor of 25(OH)D levels (biological marker of adherence) at week 48 than adherence measured by pill count. Among those given vitamin D supplement, 42% of the variation in 25(OH)D level was explained by EMD measured adherence, while only 10% of the 25(OH)D level was explained by pill count adherence, and there was no evidence that the two were associated. This suggests that EMD-based adherence monitoring more accurately reflected timely ingestion of pills than pill count. These findings align with existing evidence from other age groups that adherence measured by EMD has a better correlation with plasma drug concentrations compared to pill count or self-report methods [31–33]. For example, a study of 80 injection drug users living with HIV in China demonstrated that adherence measured by an EMD that incorporated dose-timing was more strongly associated with achieving undetectable viral loads than pill count or self-report [34].

Adherence values recorded from the EMD had a wider distribution than adherence values observed using pill count suggesting that EMD may be more sensitive as it accurately detects high and low adherers [12]. Although EMDs and pill count have a recognised limitation of not being able to confirm medication ingestion, our study findings showed that opening of the EMD equated to medication ingestion as confirmed by the biological marker [35, 36].

Explanations for discordances observed between EMD and pill count adherence included curiosity openings of the EMD [35], decanting of tablets [37, 38], as well as participants’ misunderstanding of the purpose of the EMD as a storage container. These discordances show that no adherence measurement is without limitations and reflect the need for continued information provision in ongoing adherence counselling about the reason for using the EMD and how it assesses adherence. Despite these challenges, the EMD still performed better than pill count in predicting adherence.

The EMDs sent heartbeats to the server on most days even when people were in areas with low network connectivity, and no data was lost despite some EMDs being offline for more than 2 weeks. An advantage of the EMD used in this study was its ability to store data on the device when offline and the ability to backfill that data to the server when a network connection was established [26]. The backfilled data allows patients and healthcare providers to retrospectively view adherence calendars even when the EMD is offline. Although loss of battery life has been previously reported as one of the challenges when using EMDs [39, 40], this was not observed in our study. A possible explanation is the infrequent (once per week) use of the EMDs without any other functions being activated such as text message reminders, resulting in minimal battery power required.

Overall adolescents in this study found the EMD highly acceptable. In particular, older adolescents felt that the EMD maintained privacy on their health status and medication taking as it resembled a power bank, a device widely used by adolescents and young adults in everyday settings [41]. Providing solutions to ensure privacy in medication taking is fundamental to supporting treatment adherence in adolescent health [42]. Although the EMD used in this study was compact and could easily fit in a trouser pocket, some adolescents wanted a smaller and more aesthetically appealing device. Importantly, similar findings were reported in early work on adolescent acceptability of EMDs in China [43]. This study demonstrated that real-time electronic drug monitoring was feasible and acceptable among adolescents receiving ART [43]. The participants reported few difficulties with using the EMDs and, as in our study revealed concerns about inconvenience and dissatisfaction with the EMD’s size and appearance, mirroring feedback also previously documented among adults [43, 44]. These cross-setting findings highlight the importance of ensuring that EMDs are not only functional but also discreet, portable, and visually appealing to adolescents.

Although beyond the scope of this study, the high unit cost of the EMD used (USD 200.00) might be a potential barrier to scale-up. While cost is a key component of feasibility, our primary focus was on technical and practical feasibility, such as functionality, reliability, and user acceptability. Nonetheless, the issue of cost warrants critical reflection. The current price of these EMDs far exceeds that of entry-level smartphones in resource-limited settings [45, 46], raising questions about their cost-efficiency. Future efforts should prioritize the development of lower-cost, scalable digital adherence tools [47]. Such innovations could support broader, more equitable implementation in resource-limited settings [47].

This proof of concept study contributes to understanding how EMDs can be used in a population (adolescents) and setting (resource-constrained) where the evidence base is limited [18]. It will guide future use of EMDs in this age group, including a planned intervention study using EMDs among young people living with HIV in Zimbabwe [48]. Strengths of our study include validation of EMD measures against a biological marker of adherence. The use of an explanatory sequential mixed-methods design, which enabled understanding of the quantitative findings, is also a strength. The follow-up qualitative interviews provided context to the observed adherence behaviour; for example, whether missing adherence data from the EMD was due to non-use of the device or non-adherence and offered participant-centered insights into the acceptability of the EMD. This approach strengthened the interpretation of results by linking to adolescents’ lived experiences, enhancing the practical relevance of the findings. Although the study population consisted of adolescents living with HIV, this group was not purposively selected but reflected the existing cohort enrolled in the parent VITALITY trial. As such, while findings may not be generalizable to all adolescent populations, they offer important insights into the validity, feasibility and acceptability of EMDs for supporting adherence in adolescents with chronic conditions requiring long-term medication use.

This study has several limitations. Firstly, this study assessed adherence to once-weekly vitamin D rather than a daily, lifelong therapy such as ART. Weekly dosing likely reduces the burden of pill taking and may therefore make adherence, feasibility, and acceptability appear more favorable than would be expected with daily chronic medication, where adherence patterns and barriers are often more complex [49, 50]. While this dosing schedule limits the direct applicability of findings to daily medication contexts, the sample was adequate for examining the relationship between EMD-measured adherence and 25(OH)D levels. Despite these contextual boundaries, the study provides valuable insights into the feasibility, acceptability, and performance of EMDs in a resource-limited adolescent population. The generalizability of findings may be limited, as experiences with electronic adherence monitoring could differ outside of a clinical trial context where adherence support and follow-up are more structured. In addition, interview participants may have provided overly positive reports on acceptability or adherence-related behaviors due to social desirability bias, particularly given their regular interactions with research field staff. Second, the sample size was determined by the availability of 100 EMDs. Due to the high cost of the EMDs, no additional units could be purchased, which restricted participant numbers. However, the aim of this study was not to compare adherence outcomes between groups, but to assess the validity of EMD-measured adherence through its correlation with a biological marker of adherence.

Lastly, our analysis used vitamin D levels measured at week 48 during the EMD monitoring period due to the sampling schedule of the parent VITALITY trial which could not be modified for this nested sub-study. While more frequent measurements (e.g., every 12 weeks) could have provided a more detailed picture of adherence patterns, serum 25(OH)D has a relatively long half-life of 2 to 3 weeks [51]. This pharmacokinetic property allows 25(OH)D to reflect cumulative vitamin D intake over time rather than short-term fluctuations [51, 52]. This makes 25(OH)D a suitable biomarker for evaluating medium to long-term adherence, particularly in the context of sustained weekly supplementation [52, 53]. Although short-term lapses in adherence may not be immediately detectable, the week 48 measurement remains a biologically relevant and valid indicator of overall adherence during the preceding 24 weeks.

## Conclusions

In summary, our study shows that the use of EMDs by adolescents is acceptable and feasible in Zimbabwe. We demonstrated that EMDs provide a more reliable measure of medication adherence than pill count. While the study was conducted in Zimbabwe, these findings are likely transferable to similar low-resource settings. More broadly, when implementing EMDs in adolescent populations, emphasis should be placed on providing ongoing information about the purpose of the EMDs, and on the development of affordable and discreet designs that support privacy and acceptability across diverse contexts.

## Supplementary Information

Supplementary material 1: Figure S1. Wisepill RT2000 dispenser. Supplementary figure showing the EMD that was used for this study.

Supplementary material 2: Figure S2. Example of a participant’s adherence calendar downloaded from the cloud-based server. Supplementary figure showing the adherence calendar derived from the EMD data for an individual participant. Each shaded green column represents 1 day of follow-up per week, with the black diamond inside the square indicating whether a dose was taken.

Supplementary material 3: Interview guide Semi-structured interview guide used to explore acceptability of the EMD among adolescents and reasons for discordance between adherence measured by the EMD and pill count.

Supplementary material 4: Analytical memo and qualitative codebook Combined document detailing the qualitative coding framework and code definitions, alongside reflections and decision-making processes developed during data analysis.

Supplementary material 5: COREQ checklist Completed COREQ checklist outlining how qualitative reporting criteria were addressed in this study.

## Data Availability

All the individual participant data collected during this study will be made available after de-identification, together with a data dictionary, relevant sections of the parent VITALITY trial protocol, informed consent documents, and the analytic code (Stata do-files). The dataset will be deposited in the curated repository DataCompass, and will be made available one year following publication, in line with the parent trial’s data sharing policy. As specified in the VITALITY protocol, this embargo period allows time for completion of prespecified sub-analyses by study staff and research degree students and ensures compliance with national ethics committee requirements. Data will thereafter be available indefinitely. Investigators who wish to use the data may request access via DataCompass https://datacompass.lshtm.ac.uk. Approval from national ethics committees in Zimbabwe will be required for data usage, and data requestors will need to sign a data sharing agreement.

## Abbreviations

25(OH)D: 25 Hydroxy vitamin D
95% CI: 95% Confidence interval
ART: Antiretroviral therapy
EMDs: Electronic monitoring devices
HIV: Human immunodeficiency virus
IQR: Interquartile range
VITALITY: VITamin D for AdoLescents with HIV to reduce musculoskeletal morbidity and ImmunopaThologY
