# Supplementary figures and images for: Feasibility, acceptability and validity of electronic adherence monitoring among adolescents in Zimbabwe: a mixed methods study
Source: BMC Glob Public Health. 2026 Feb 10;4:17. doi: 10.1186/s44263-026-00248-z (PMC12892711; doi:10.1186/s44263-026-00248-z)

**Figure S1: Wisepill RT2000 dispenser**
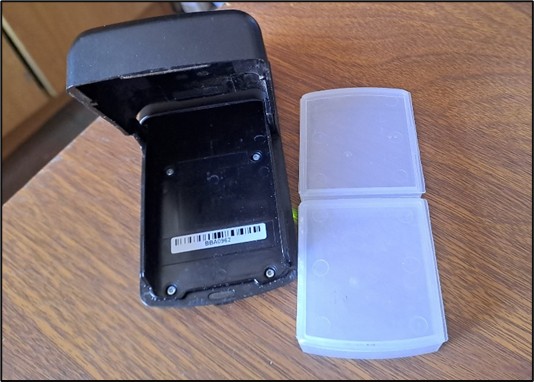

Supplement: Supplementary file 1 — Supplementary material 1: Figure S1. Wisepill RT2000 dispenser. Supplementary figure showing the EMD that was used for this study. [file 44263_2026_248_MOESM1_ESM.docx]

**Figure S2: Example of a participant’s adherence calendar downloaded from the cloud-based server**


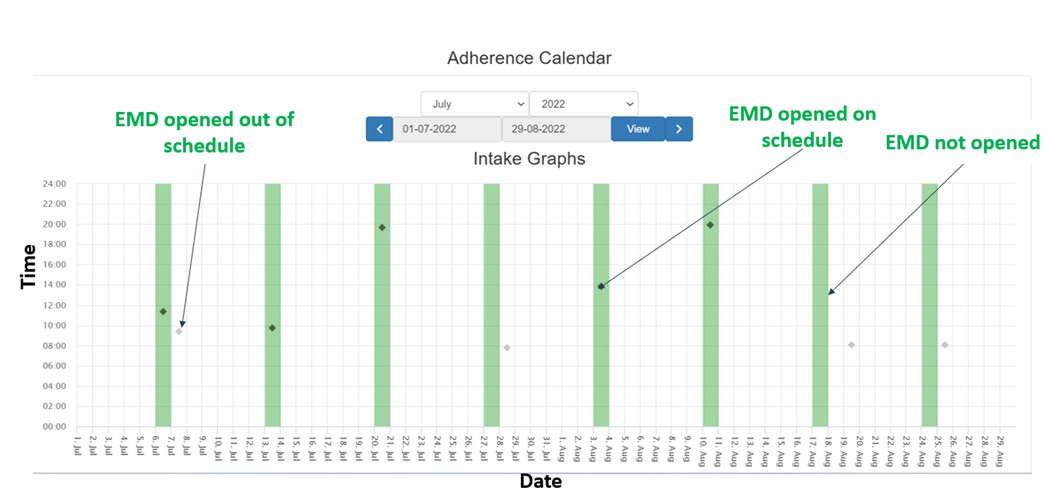

Supplement: Supplementary file 2 — Supplementary material 2: Figure S2. Example of a participant’s adherence calendar downloaded from the cloud-based server. Supplementary figure showing the adherence calendar derived from the EMD data for an individual participant. Each shaded green column represents 1 day of follow-up per week, with the black diamond inside the square indicating whether a dose was taken. [file 44263_2026_248_MOESM2_ESM.docx]
