# Supplementary material for: Feasibility, acceptability and validity of electronic adherence monitoring among adolescents in Zimbabwe: a mixed methods study
Source: BMC Glob Public Health. 2026 Feb 10;4:17. doi: 10.1186/s44263-026-00248-z (PMC12892711; doi:10.1186/s44263-026-00248-z)
Supplement: Supplementary file 3 — Supplementary material 3: Interview guide Semi-structured interview guide used to explore acceptability of the EMD among adolescents and reasons for discordance between adherence measured by the EMD and pill count. [file 44263_2026_248_MOESM3_ESM.docx]

Interviewer: This device gave us information every week of how well you were taking your medication. We are going to be looking at your graph so that we can discuss how well you did with taking your vitamin D tablets in the last 2 months (shows participant their adherence graph on the server).

The black diamonds (point to the screen and show participant on the server) show the number of times you opened the device to take your vitamin D on the day you were supposed to take it (mention day to participant).

1. Can you please tell me what you see on the graph?
   Probe on the number of times the participant thinks they opened the pillbox – make sure the child understands what is shown on the graph. Spend some time until you are convinced participant understands how the graph Sense 3.0 platform was recording events.

If adherence data shows correct pill intake as per schedule events, ask

1. It looks like you took your vitamin D at the correct time every week– well done. What made it easy to take your vitamin D on these weeks?
2. Probe on the ease of using the pill box – i.e., opening and closing the box, if there was someone at home reminding the participant to take their tablets or whether there was use of an alarm
3. Probe on whether participant felt the device was discrete, comfortable with using it around other people
4. Explore if any, the reason for discrepancies with their week 48 visit pill count that is participant brought back more or less than the expected number of vitamin D tablets – explore whether they had extra tablets from the previous visit (*can verify with week 36 visit pharmacy form)* or took more than the required dose (1 tablet once weekly) ***^*^NB^*^ if participant comes back on scheduled week 48 visit they should bring back at least 3 or 4 vitamin D tablets and 29 or 30 calcium tablet. If visit was out of schedule i.e., before scheduled week 48 visit number of vitamin D brought back should be more than 3 or 4, if after scheduled week 48 visit number of vitamin D tablets brought back should be less than or 3 or 4.***
5. If participant brought back the correct number of vitamin D tablets consistent with pill box opening events on the Sense platform but incorrect calcium tablets – explore the reasons for this discrepancy and check whether if using pillbox for calcium would have helped improve calcium adherence
6. Explore if any, the reasons for extra or out of range pill box opening events as shown on the Sense platform

If adherence data shows consistent out of range pill intake events, ask

1. There were weeks when you did not take your vitamin D correctly (point at the out of events schedules). What made it difficult for you to take your vitamin D in those weeks?
2. Probe on the ease of using the pill box – i.e., opening and closing the box, if there was someone at home reminding the participant to take their tablets or whether there was use of an alarm.
3. Probe whether the participant knew/ understood when to take their vitamin D tablets as explained by VITALITY study staff (***ask the participant to repeat how they were taking their vitamin D tablets at home)***
4. Probe whether participant felt the device was intrusive, if there were times that prevented participant from opening the pillbox i.e., not at home, or comfort with using the pill box around other people at home.
5. Explore if any, the reason for discrepancies with their week 48 visit pill count i.e., brought back more or less than the expected number of vitamin D tablets for week 48 visit. Ask if the participant had extra tablets from their week 36 visit ***(verify with week 36 pharmacy form)*** or if they took more than the required tablets for that week. ***(allow participant to explain)*** ***-*NB* if participant comes back on scheduled week 48 visit, they should bring back at least 3 or 4 vitamin D tablets and 29 or 30 calcium tablet. If visit was out of schedule i.e., before scheduled week 48 visit number of vitamin D brought back should be more than 3 or 4, if after scheduled week 48 visit number of vitamin D tablets brought back should be less than or 3 or 4.***
6. Explore if any, the reasons for extra pill opening box events as shown on the Sense platform.

If adherence data shows more than 75% of no pill box opening events, ask

1. There were weeks when you did not open the device to take your vitamin D tablets (point at the non-opening event schedules). What made it difficult for you to take your vitamin D tablets in those weeks?
   1. Probe on the ease of using the pill box – i.e., opening and closing the box, if there was someone at home reminding the participant to take their tablets or whether there was use of an alarm.
   2. Probe whether the participant knew/ understood when to take their vitamin D tablets as explained by VITALITY study staff (***ask the participant to repeat how they were taking their vitamin D tablets at home)***
   3. Probe whether participant felt the device was intrusive, if there were times that prevented participant from opening the pillbox i.e., not at home, or comfort with using the pill box around other people at home.
   4. Explore if any, the reason for discrepancies with their week 48 visit pill count i.e., if participant brought back the correct number or fewer than expected vitamin D tablets inconsistent with what is shown on the Sense 3.0 platform probe for reasons for this discrepancy – ask whether they removed pills from the pill box and stored them elsewhere – if yes ask participant to explain why.

****NB* if participant comes back on scheduled week 48 visit, they should bring back at least 3 or 4 vitamin D tablets and 29 or 30 calcium tablet. If visit was out of schedule i.e., before scheduled week 48 visit number of vitamin D brought back should be more than 3 or 4, if after scheduled week 48 visit number of vitamin D tablets brought back should be less than or 3 or 4.***

- 1. Explore if any, the reasons for extra or out of range pill opening box events as shown on the Sense platform.

If adherence data shows a mixed picture good events recorded and no events recorded ask question 5

1. What made it easier to take your vitamin D in some weeks (show the participant the good weeks) compared to these weeks (point to the times when the participant did not open the device)?
   1. Probe on the ease of using the pill box – i.e., opening and closing the box, if there was someone at home reminding the participant to take their tablets or whether there was use of an alarm.
   2. Probe whether the participant knew/ understood when to take their vitamin D tablets as explained by VITALITY study staff
   3. Probe whether participant felt the device was intrusive during the weeks that they did not open the pillbox, if there were times that prevented participant from opening the pillbox i.e., not at home, or comfort with using the pill box around other people at home
   4. Explore if any, the reason for discrepancies with their week 48 visit pill count i.e., if participant brought back the correct number or fewer than expected vitamin D tablets inconsistent with what is shown on the sense 3.0 platform probe for reasons for this discrepancy – ask whether they removed pills from the pill box and stored them elsewhere – if yes ask participant to explain why.

**NB* if participant comes back on scheduled week 48 visit, they should bring back at least 3 or 4 vitamin D tablets and 29 or 30 calcium tablet. If visit was out of schedule i.e., before scheduled week 48 visit number of vitamin D brought back should be more than 3 or 4, if after scheduled week 48 visit number of vitamin D tablets brought back should be less than or 3 or 4.*

- 1. Explore if any, the reasons for extra or out of range pill opening box events as shown on the Sense platform.

All participants

1. What were you usually doing during the time that you are supposed to take your vitamin D tablets? Did what you were doing during the time that you were supposed to take your vitamin D tablets make it easy or difficult for you to take your vitamin D tablets?
2. Is there anything that would have made it easier for you to use this device so that you do not miss to take your vitamin D tablets?
3. Do you think using the Wisepill pill box would make it easier for you to remember to take your ARV tablets on time? Explain.
4. If we were to give the Wisepill pill box to other young people like you to use to put their ARV tablets, do you think they will agree or accept to use the pill box? Explain.
5. Is there anything else that you would like to tell me about how you were using the pill box?

Thank the participant for using the Wisepill RT2000 device and answering the exit interview questions.
