# Supplementary material for: Feasibility, acceptability and validity of electronic adherence monitoring among adolescents in Zimbabwe: a mixed methods study
Source: BMC Glob Public Health. 2026 Feb 10;4:17. doi: 10.1186/s44263-026-00248-z (PMC12892711; doi:10.1186/s44263-026-00248-z)
Supplement: Supplementary file 4 — Supplementary material 4: Analytical memo and qualitative codebook Combined document detailing the qualitative coding framework and code definitions, alongside reflections and decision-making processes developed during data analysis. [file 44263_2026_248_MOESM4_ESM.docx]

**Additional file 4: Vitamin D pilot analytical memo and codebook**

***Objectives***

1. To explore acceptability of EMDs
2. To investigate the reasons for discordance in EMD recorded adherence and pill count

Research questions that emerged from the data

1. Are EMDs acceptable to use in adolescents living with HIV?
2. What are the reasons for discordance in EMD recorded adherence and pill count?

**The process**

1. Familiarisation – read all transcript multiple times to get an understanding of what participants were saying and wrote down initial ideas of what participants were saying.
2. Coding done in NVivo-14 – initially deductive coding was done based on the following two research questions.
   1. Are EMDs acceptable to use in ALWH?
   2. What were the reasons for discordance between EMD recorded adherence and pill count
3. All transcripts coded using the codes developed and any new other emerging codes
4. Exported codes into an excel spreadsheet to categorise the codes and develop themes. Godes that spoke towards the same thing were put into the same category – to create a common theme
5. Themes reviewed (multiple times/iteratively) by
   1. Going through the coded data in each theme to check for coherence
   2. Checking the themes to see if they accurately represent the meanings in the dataset as a whole
6. Themes were also re-defined and renamed during the reviewing process – involved looking at what aspect of the data the theme is capturing
7. Interpretation and producing of the report – reporting the findings from the interviews to tell a story
8. A code book with main theme – sub-themes and codes associated with the theme are listed below (summary of code book presented below on page 2 and 3)

| **Theme** | **Definition of theme** | **Sub-themes** | **Codes associated with theme** |
| --- | --- | --- | --- |
| Acceptability of EMDs | Theme explored how satisfied in terms of ease of use, storage of medication, any benefits that were associated with using the EMD as well as any challenges that participants faced because of using the pill box. | ***Ease of use of EMD*** | - EMD easy to use - No problems with using the EMD - Instruction video helpful |
|  |  | ***Safe storage for medication*** | - EMD keeps medication safe |
|  |  | ***Discreet*** | - Comfortable with using EMD around people - Easy to move around with - EMD was discreet |
|  |  | ***Help improve adherence*** | - Good for adherence monitoring - Good reminder to take pills - Allows one to follow a structured pattern of adherence |
|  |  | ***Perceived stigma with using the pill box*** | - Could not use the device around people - Difficult to move around with the device |
|  |  | ***Challenges to acceptability*** | - EMD should be smaller - Putting a reminder on the EMD |
| Reasons for discordance between EMD recorded adherence and pill count | Theme explored the reasons why some participants would have multiple or very few on schedule pill box openings that did not agree with the number of vitamin D tablets that they were supposed to take or bring back at the end of their week 48 visit. | ***Multiple openings of EMD*** | - Checking if device was still working - Other people’s fascination with the device |
|  |  | ***Had extra tablets at home*** | - Had excess tablets |
|  |  | ***Taking more tablets than prescribed*** | - Compensating by overdosing |
|  |  | ***Taking pills on a day that is not a scheduled intake day*** | - Took pills from EMD on a different day to the scheduled day |
|  |  | ***Did not use the EMD*** | - Did not understand the purpose of the pill box Misplaced tablets - Taking pills from elsewhere |

**Quotes associated with the different themes**

***Acceptability of EMDs***

Overall, all adolescents were satisfied with using the EMD and none of the adolescents experienced any challenges with using it.

**Ease of use:** All participants expressed that the EMD was easy to use. There was no one who struggled with opening or closing the EMD. Some participants went on further to express that the instruction video that they had been shown at the time of enrolment into the study had helped them to see how to use EMD.

*“It was easy to use the device.* *It was never a problem to open and close the device.”* (Q1)

*“There was a time you gave us a tablet to watch the video on how to use the pill box, so I understood there (how to use the device) when I was watching the video.”* (Q2)

***Safe for storage of medication*:** Most participants felt that the EMD helped keep their medication safe, by protecting the pills from any breakages even if it was dropped on the ground, and the fact that the EMD was airtight also helped keep the pills safe from environmental exposures such as “air or wind”.

*“…On safekeeping it was alright, it was alright because even if it falls, the pills will not come out or break, because there is a time when it fell, and I thought it could have damaged the pills inside, but they were not broken,”* (Q3)

*“I can say that it helped me because my pills were in a safe place … the container was tightly closed… and my pills were not affected by air or wind or other things and that is what always reassured me”* (Q4)

**Discreet:** Most participants, especially the older adolescents (16 years and above) felt that the EMD was useful in hiding their pills away from prying and inquisitive eyes. They also mentioned that the compactness and shape of the EMD which could easily make it pass as a power bank made it easier for them to carry around and maintain their privacy in terms of taking pills.

*“It was helpful in hiding the pills because many people did not know that the device had pills in it. “*(Q5)

*“The pill box was not embarrassing to use around people at all because when I was in my rural area people were thinking it was a power bank”* (Q6)

***Help improve adherence***: Most participants expressed that the EMD was useful for adherence monitoring especially the adherence calendar as it would help provide a visual picture of an individual’s adherence behaviour at home.

*“This graph showed me that sometimes I would forget, and at times take and not take my medication, so it then shows us what we were doing, it shows the exact things even if you are a person who lies about your medication intake.”* (Q7)

Participants went on further to express how the perception of being monitored also helped improve their adherence.

*“The good thing about this device is the fact that it records you, pushes you to drink your medication because I would say to myself, if I skip today then it means they will see me… so because they would see me, I will then go and chew my tablet.”* (Q8)

Participants also expressed that the fact that they had the EMD which was a different pillbox from the rest of their pill containers served as a reminder to take their medication.

*“Because I had the device… even if I forget to take my medication but when the time passes, I will remember that I have not opened the device… and I would then go and open it. “*(Q9)

**Perceived stigma with using the EMD:** There were a few participants who expressed that they were not comfortable with using the EMD around people as it would attract attention mostly because of the light that flashes on the side when the device is opened.

*“The appearance of this device, because you open it, then a light turns on so people would really question that, how come it lights? What is the box for? So, in public, you can’t take it out, because people may not understand you see… and they will end up seeing your pills”* (Q10)

Other participants also felt that they could not move around with the EMD because of its size and could not be wrapped in a tissue like what they usually do with pills taken from a conventional container.

*“I am saying no because you can put your pills in the device, but you are not supposed to remove the pills from the box so for you to carry the box when travelling or to going work it’s something not easy to carry, its big ... With the other containers you take a tissue, and you wrap around your pills, and you carry them around but then that device is very big and difficult in that regard.”* (Q11)

**Reasons for discordance between EMD recorded adherence and pill count**

We also explored the reasons why some participants would have multiple or very few on schedule pill box openings that did not agree with their pill count (expected number of tablets that they were supposed to bring back).

**Multiple openings of the pill box:** Some participants highlighted that the reason why they would open the EMD many times was so that they could check if the device was still working well and did not have a low battery (red light flashing) since they were only using it once a week.

*“At times when I would open when I am home, I would want to see if the red light is on, so I would open, if I see its still green then I know its ok. Then I would store the device, so as time passes if I skip about 4 or 5 days I open then see if the red light is not on”* (Q12)

Other participants reported that some of the out of schedule pill box opening events were as a result of their family members who opened the EMD as they wanted to understand what the EMD was and what was inside it.

*“On the issue of opening the device outside my pill taking day, I can say that in our house I live with my younger siblings sometimes, as we were seeing on the graphs where I opened the box outside the days, my siblings after seeing the box they started liking…Sometimes they would see me opening it* (the EMD) *taking the pills so they wanted to test the box as they were fascinated with the box and what was happening … Yes, they are the ones who opened it because they were having fun with how the pill box was working and the light that came on when the device was opened and maybe also the pills themselves, I do not know maybe they were tasting if the pills were sweet, I am not sure what they were doing after opening the box…”* (Q13)

*“Ummm it was not me opening the device, it was my Uncle who opened the device… he wanted to see what it was.”* (Q14)

**Had extra tablets at home:** A few participants mentioned that they had extra tablets at home prior to being enrolled in the study which they had to finish first before using the ones in the EMD. This is the reason why they had fewer on schedule pill box opening events.

*“Before we started there were other ones which were not put in the device we had left at home, so I was told to finish those ones before I use the device … and I don’t remember how many these extra tablets were.”* (Q15)

**Taking more tablets than prescribed:** Other participants highlighted that they would forget to open their EMD for several weeks, and then when they remembered they would take many tablets at once to compensate for the weeks that they had missed opening the EMD.

*“Most of the time when I finally remember, I would take three pills. I did this several times i.e., to open the box say after three weeks of not taking the vitamin D and then I would take three or more pills at once. Four tablets were the highest number of tablets I took all at once… and sometimes I would just think that I might have skipped two times I would have forgotten because I would not have counted the days, but I could just feel that it has been long since I opened the box and would just take many pills all at once.”* (Q16)

*“(laughing) yah yah I may have covered for the time I did not take the tablets ... I covered for the weeks that I had missed taking the tablets”* (Q17)

**Taking pills on a day that is not a scheduled intake day:** Sometimes the participants would forget to open their EMD on their scheduled intake day and would then open it a few days later after they realised that they had not taken their pill. Most of the participants who did this mentioned that they had been advised at the study clinic that if they forgot to take their pill on the scheduled intake day, they could take it on any other day as soon as they remembered.

*“At times if I forget on Friday, I would take them on Saturday.”* (Q18)

*“…there is this day when I went to ABXY, I was supposed to drink it that day, but we came back the following day, so the following day I just came and took and chewed the tablet…”* (Q19)

*“…those are the days I said I would forget, let’s say I forget today it will be on a Monday I would have missed [uhuh] I would drink either on a Tuesday or Wednesday but knowing that my day of taking I would have missed.”* (Q20)

*“**The problem is about electricity, so because of that problem, she takes her pills in the evening after coming back from school if there is no electricity she just goes into her blankets and sleep. I had to ask her to wake up and open the box, but she would not. The one who confused her is the person who told her that if you do not take the pills on time, Saturday is okay. They said Friday and they told me that if I fail on a Friday, I could take the pills Saturday in the evening”* (Q21)

**Did not use the EMD:** Other participants just did not use the EMD, and this is the reason why they recorded very few pill box opening events on the adherence calendar but had consumed the correct number of tablets or brought back the correct number of tablets to the study clinic.

Some participants did not use the EMD because they did not understand the purpose of the pill box as they felt that it was only a device for storing tablets and so they took out the plastic pill container from the EMD, set it aside and took their pills from the plastic pill container.

*“I did not understand what the purpose of the box was, I thought that it was something to just store my pills. You See, what was happening on that box is that when I got it I thought it was something for me to store my pills like our trays we use to keep the pills, so I thought it was the same using the box and putting the pills on the tray… so I opened the device three or four times if I am not mistaken.”* (Q22)

Other participants failed to use the EMD because they had misplaced it.

*“I can say it is a period when we relocated, I could not see my tablets and many other things even my books which I used to collect my pills, I found them later and when I came here, they asked why I was not opening the box and I told them that I had misplaced my things…”* (Q23)

Other participants highlighted that while they did not fail to take their pills religiously however, they opted to take their pills from elsewhere. This mostly happened to participants who would have travelled from their home and would leave the EMD behind.

*“I did not fail to take my pills, but I opened the box at home and took the pills with me to EXTY. I did not go with the box when I went to EXTY. I left it at home. I just opened the pill box to take the pills that were enough for the duration of my stay in EXTY.* (Q24)

*“From the tray those are the ones I was using … Yes, and I think that is where the error happened... because I was using pills from elsewhere… I had pills, the ones on the tray these are the ones I was using”* (Q25)
